# Complete and Incomplete Genome Packaging of Influenza A and B Viruses

**DOI:** 10.1128/mBio.01248-16

**Published:** 2016-09-06

**Authors:** Sumiho Nakatsu, Hiroshi Sagara, Yuko Sakai-Tagawa, Norio Sugaya, Takeshi Noda, Yoshihiro Kawaoka

**Affiliations:** a Division of Virology, Department of Microbiology and Immunology, Institute of Medical Science, University of Tokyo, Tokyo, Japan; b Medical Proteomics Laboratory, Institute of Medical Science, University of Tokyo, Tokyo, Japan; c Department of Pediatrics, Keiyu Hospital, Kanagawa, Japan; d PRESTO, Japan Science and Technology Agency, Saitama, Japan; e Department of Pathobiological Sciences, School of Veterinary Medicine, University of Wisconsin—Madison, Madison, Wisconsin, USA; f International Research Center for Infectious Diseases, Institute of Medical Science, University of Tokyo, Tokyo, Japan

## Abstract

The genomes of influenza A and B viruses comprise eight segmented, single-stranded, negative-sense viral RNAs (vRNAs). Although segmentation of the virus genome complicates the packaging of infectious progeny into virions, it provides an evolutionary benefit in that it allows viruses to exchange vRNAs with other strains. Influenza A viruses are believed to package their eight different vRNAs in a specific manner. However, several studies have shown that many viruses are noninfectious and fail to package at least one vRNA. Therefore, the genome-packaging mechanism is not fully understood. In this study, we used electron microscopy to count the number of ribonucleoproteins (RNPs) inside the virions of different influenza A and B virus strains. All eight strains examined displayed eight RNPs arranged in a “7+1” configuration in which a central RNP was surrounded by seven RNPs. Three-dimensional analysis of the virions showed that at least 80% of the virions packaged all eight RNPs; however, some virions packaged only five to seven RNPs, with the exact proportion depending on the strain examined. These results directly demonstrate that most viruses package eight RNPs, but some do indeed package fewer. Our findings support the selective genome-packaging model and demonstrate the variability in the number of RNPs incorporated by virions, suggesting that the genome-packaging mechanism of influenza viruses is more flexible than previously thought.

## IMPORTANCE

The genomes of influenza A and B viruses contain segmented RNAs, which complicates genome packaging but provides the evolutionary advantage of allowing the exchange of individual genome segments with those of other strains. Some studies have shown that influenza A viruses package all eight genome segments in a specific manner, whereas others have shown that many virions are noninfectious and fail to package at least one genome segment. However, such viruses have never been directly observed. Here, we used electron microscopy to provide the first direct visual evidence of virions packaging an incomplete set of ribonucleoproteins. The percentage of these noninfectious virions varied from 0 to 20, depending on the virus strain, indicating that most virions package all eight genome segments. These results extend our knowledge about how infectious and noninfectious virions coordinate for successful virus infection.

## INTRODUCTION

Influenza A and B viruses, which belong to the family *Orthomyxoviridae*, have a genome consisting of eight single-stranded, negative-sense RNAs. Each viral RNA (vRNA) segment, which encodes a different viral protein(s) essential for efficient virus replication, differs in length, ranging from 890 to 2,341 bases (1). Each vRNA segment binds to an RNA-dependent RNA polymerase complex and multiple copies of a nucleoprotein to form a helical, rod-like ribonucleoprotein (RNP) (2–4). All RNPs have a uniform diameter of approximately 12 nm but differ in length, ranging from approximately 30 to 120 nm, depending on the nucleotide length of each vRNA (5).

The segmented vRNAs allow influenza viruses to rapidly evolve via genome reassortment, which is thought to be responsible for the emergence of pandemic influenza viruses such as the pandemic A (H1N1) 2009 virus strain (6–8). However, segmentation of the genome complicates the genome-packaging process, because all eight different vRNAs must be packaged into each virion to produce infectious progeny. Recent studies support the selective packaging model, in which a virion incorporates the eight distinct RNPs via specific mechanisms that are not yet fully understood (9, 10). Segment-specific packaging signals that drive the efficient incorporation of the resident vRNAs into the virion have been identified at both the 3′ and 5′ ends of all vRNA segments (11–19). Both terminal coding regions within the packaging signal act as a bundling signal to ensure that all eight distinct vRNAs are incorporated (20). When viewed with an electron microscope, the eight RNPs are usually observed within the virions in a characteristic “7+1” configuration in which a central RNP was surrounded by seven RNPs (21–24). The presence of eight distinct vRNAs in most virions examined has also been demonstrated by single-molecule fluorescent *in situ* hybridization analysis (25). In addition, *in vitro* vRNA-vRNA interactions between two different vRNA segments have been shown by using native agarose gel electrophoresis, and the regions for inter-vRNA interactions have been shown to be important for efficient virus replication (24–26). Collectively, these findings suggest that influenza A viruses use selective packaging of a complete set of eight vRNAs as a mechanism to ensure the production of fully infectious virions.

However, some studies have shown that the virus particle-to-plaque-forming unit (PFU) ratio for influenza A viruses is greater than 10:1, suggesting that most virus particles are defective in their vRNA packaging (27, 28). The existence of noninfectious viruses has also been suggested by the observation of A/WSN/33 (A/WSN) virus-infected cells that fail to express some viral proteins (29). A recent study using flow cytometry demonstrated that almost half of A/Puerto Rico/8 (A/PR8) virus-infected cells failed to express one or more essential viral proteins, suggesting that nearly half of all influenza A viruses are semiinfectious (SI) virions. These SI virions might fail to express some viral proteins essential for the replication cycle, but nonetheless, they are able to produce progeny by complementing the absent proteins through coinfection (30). In addition, the presence of such SI virions is considered to enhance genome reassortment in coinfected cells (31). The authors of these previous studies have generally assumed that the lack of viral protein expression is exclusively due to the failure of vRNA packaging into virions, proposing that virions containing a complete set of RNPs would be in the minority. However, loss of viral protein expression could also be due to a breakdown in the uncoating and nuclear transport of RNPs, vRNA transcription, and/or viral protein synthesis (32). Given that SI virions have never been directly observed, the existence of virions containing less than eight RNPs remains controversial.

Previous studies demonstrating that influenza A viruses selectively package eight RNPs arranged in a specific “7+1” pattern relied on electron microscopic techniques and mainly used laboratory strains of influenza A viruses, which are well adapted to cell culture or embryonated eggs (22, 24). In fact, quantitative analyses of vRNP packaging have not been done even with laboratory strains. To reveal whether virions incorporating less than eight RNPs actually exist and, if they do exist, to determine what proportion of virions contain less than eight RNPs, we counted the number of RNPs in both laboratory and clinically isolated strains of influenza A and B viruses by using ultrathin-section electron microscopy and electron tomography.

## RESULTS

### Genome packaging of influenza A viruses.

**FIG 1:**
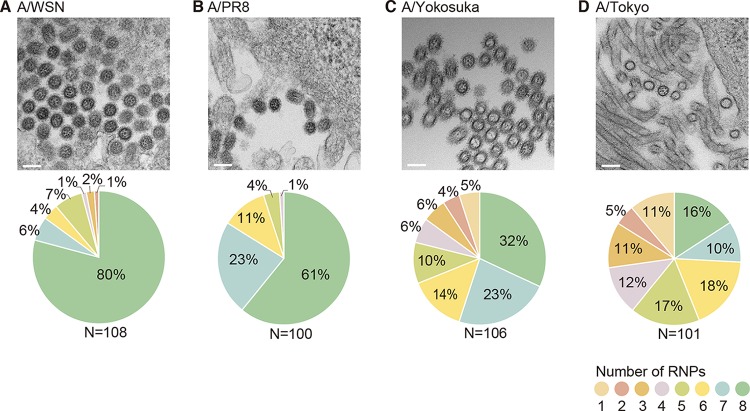
RNPs observed within transverse sections of influenza A virions. MDCK cells were infected with A/WSN/33 (A/WSN) (A), A/Puerto Rico/8 (A/PR8) (B), A/Yokosuka/UT-Y291/11 (A/Yokosuka) (C), and A/Tokyo/UT-IMS6-1/2013 (A/Tokyo) (D) at an MOI of 10. At 48 h postinfection (hpi), the infected cells were fixed and embedded in Epon resin. Ultrathin sections (110 nm thick) were prepared and observed by using TEM. The pie charts show the proportions of virions in which one to eight RNPs were observed. Scale bar, 100 nm.

To investigate whether virions from various influenza A virus strains package all eight well-organized RNPs, we prepared 110-nm-thick ultrathin sections of the budding virions of four influenza A virus strains (A/WSN and A/PR8 as representative laboratory strains and A/Yokosuka/UT-Y291/11 [A/Yokosuka] and A/Tokyo/UT-IMS6-1/2013 [A/Tokyo] as representative clinically isolated strains) and counted the number of RNPs observed within the virions by using transmission electron microscopy (TEM). Because influenza virions are spherical or oval, with a diameter of approximately 100 nm (1), we expected to be able to count most of the RNPs packaged within the virions with a high probability by using the 110-nm-thick ultrathin sections. As previously reported (21–24), most A/WSN virions (80%) contained eight RNPs arranged in the “7+1” pattern in which a central RNP was surrounded by seven RNPs (Fig. 1A). However, virions containing less than eight RNPs (i.e., seven, six, five, four, three, two, or one RNP in each sectioned virion) were also observed.

Well-organized sets of eight RNPs were also observed within the virions of the other influenza A virus strains, although the proportions of such virions differed, depending on the virus strain (Fig. 1). Approximately 60% of the A/PR8 virions had all eight RNPs (Fig. 1B), whereas only 32% and 16% of the virions from the clinical isolates A/Yokosuka and A/Tokyo, respectively, contained all eight RNPs, (Fig. 1C and D). These results suggest that both laboratory and clinically isolated influenza A virus strains use a common mechanism to package the eight RNPs and arranged them in a “7+1” pattern, although clinically isolated strains appeared to show less-efficient packaging of the eight RNPs.

### Genome packaging of influenza B viruses.

**FIG 2:**
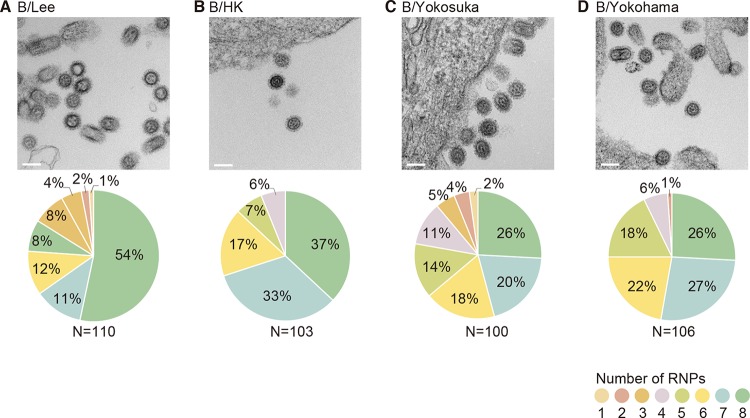
RNPs observed within transverse sections of influenza B virions. MDCK cells were infected with B/Lee/40 (B/Lee) (A), B/Hong Kong/8/73 (B/HK) (B), B/Yokosuka/UT-Y23/11 (B/Yokosuka) (C), and B/Yokohama/UT2086/03 (B/Yokohama) (D) at an MOI of 10. At 48 h postinfection (hpi), the infected cells were fixed and embedded in Epon resin. Ultrathin sections (110 nm thick) were prepared and observed by using TEM. The pie charts show the proportions of virions in which one to eight RNPs were observed. Scale bar, 100 nm.

Most studies of the genome-packaging mechanism of influenza viruses have focused on influenza A viruses, and little is known about the genome-packaging mechanism of influenza B viruses, although these viruses also possess genomes with eight-segmented RNAs. To determine whether influenza B viruses package their eight-segmented RNPs into virions, 110-nm-thick ultrathin sections of budding virions were prepared from four influenza B virus strains (the laboratory strains B/Lee/40 [B/Lee] and B/Hong Kong/8/73 [B/HK] and the clinically isolated strains B/Yokosuka/UT-Y231/11 [B/Yokosuka] and B/Yokohama/UT2086/03 [B/Yokohama]). Then, the number of RNPs observed within transversely sectioned virions of each strain was counted manually by using TEM as described for the influenza A viruses. The characteristic “7+1” configuration of eight RNPs was observed in all of the influenza B strains examined (Fig. 2A to D). All eight of the well-organized RNPs were observed in about 50% of B/Lee virions and in about 40% of B/HK virions. However, as with the influenza A viruses, the proportions of virions containing eight RNPs tended to be lower for the clinically isolated strains: 26% each for B/Yokosuka and B/Yokohama (Fig. 2A to D). These findings imply that although a substantial number of virions package less than eight RNPs, influenza A and B viruses have retained the ability to package all eight well-organized RNPs.

### Scanning transmission electron microscopy tomography of influenza A and B virions.

The results of the TEM analysis of ultrathin sections of influenza A and B virions suggest that incomplete packaging of RNPs into a substantial number of virus particles occurs, with variation among virus strains. However, because whole virions are not always contained within 110-nm-thick ultrathin sections, the number of RNPs observed within the sectioned virions might not necessarily represent the actual number of RNPs within the virions; therefore, these results do not conclusively demonstrate whether the virions package less than eight RNPs (see Fig. S1 in the supplemental material). To determine the exact number of RNPs packaged within a particular whole virion, we performed scanning transmission electron microscopy (STEM) tomography for the A/WSN, A/Yokosuka, B/Lee, and B/Yokosuka strains. For this analysis, 250-nm-thick semithin sections of budding virions were subjected to STEM tomography as described previously (22), and only whole virions, that is, not sectioned virions, were three-dimensionally (3-D) reconstructed. Then, the number of RNPs within each whole virion was counted using the 3-D information.

**FIG 3:**
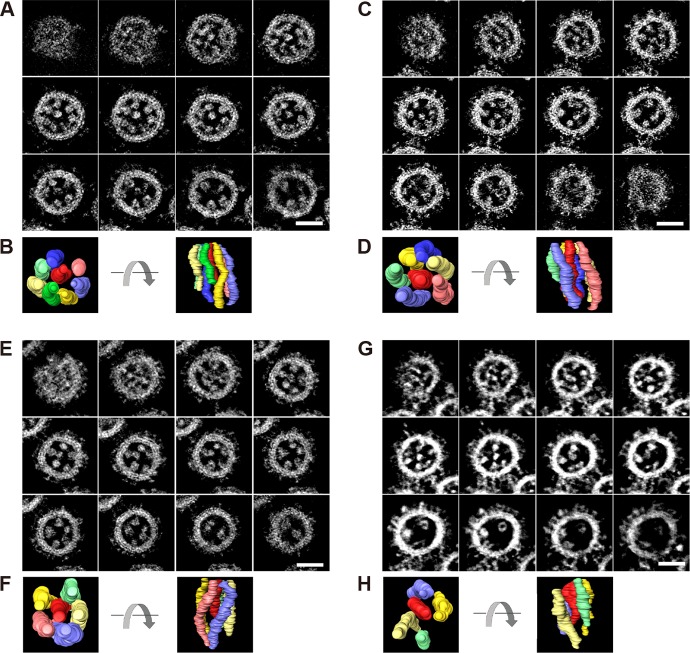
RNPs within 3-D reconstructed virions. For each virus strain, 250-nm-thick semithin sections were prepared from the same samples as those examined by using TEM (Fig 1 and Fig 2). Then, 3-D structures of the whole virions were computationally reconstructed by using STEM tomography. (A and B) A/WSN, (C to F) B/Lee, and (G and H) A/Yokosuka. Digital slices of the reconstructed virions for A/WSN (A), B/Lee (C and E), and A/Yokosuka (G) are shown from the top (top left panel) to the bottom (bottom left panel). (B, D, F, and H) Models for the RNPs packaged within the virions from the top (right) and side (left) views. Scale bar, 75 nm.

Consecutive 0.5-nm-thick tomograms obtained at 5.0-nm intervals from the respective whole virions demonstrated that different lengths of the eight RNPs, associated with the lipid envelope at the top of the budding virion, were frequently found within a single whole virion in all influenza A and B strains examined, confirming the results of a previous study (22) (Fig. 3A; see Fig. S2C and S2D in the supplemental material). A 3-D model of RNPs within a single whole virion (created based on the electron density of RNPs in consecutive tomograms) clearly showed that the eight RNPs were consistently arranged in the “7+1” pattern in all strains, demonstrating the importance of this well-organized supramolecular complex for the complete packaging of the eight vRNAs in both influenza A and B viruses.

However, some virions were found to have packaged less than eight RNPs (Fig. 3C, E, and G). For example, seven RNPs were found packaged in one virion (Fig. 3C); these RNPs were associated with the inner envelope of the budding virion at the top, similar to the observations of the well-organized packaging of all eight RNPs. The seven RNPs were arranged in a “6+1” pattern, in which a central RNP was surrounded by six RNPs. Similarly, six RNPs arranged in a “5+1” pattern were found in another virion (Fig. 3E). In addition, we observed occasional virions containing only five RNPs organized in a “4+1” arrangement (Fig. 3G). No virions were found to package more than eight RNPs, although thin actin-like structures were observed in A/Yokosuka (see Movie S1D in the supplemental material). These results confirm our finding that some virions package less than eight RNPs and that incomplete genome packaging of the eight vRNAs occasionally occurs in both influenza A and B virions, resulting in the production of virions with less than eight RNPs.

### Influenza A and B virions mostly package eight RNPs.

**FIG 4:**
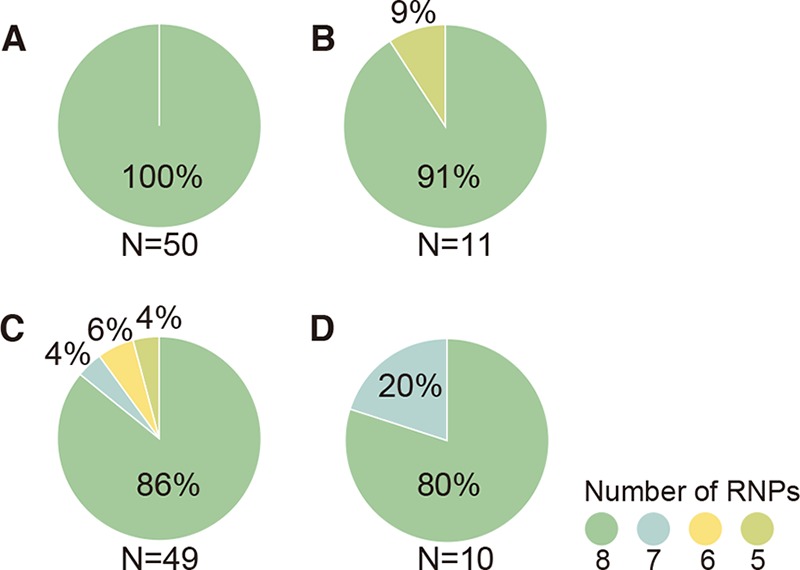
Influenza A and B viruses usually package eight RNPs. The number of RNPs inside the virions was counted for each reconstructed virus acquired by use of STEM tomography. Approximately 50 virions of the laboratory strains and 10 virions of the clinically isolated strains were analyzed. (A) A/WSN, (B) A/Yokosuka, (C) B/Lee, and (D) B/HK.

Within a viral population, what proportion of virions package the complete set of RNPs? To quantitatively determine the proportion of budding virions that package all eight RNPs, approximately 50 whole virions of laboratory strains and 10 whole virions of clinically isolated strains were 3-D reconstructed by using STEM tomography, and the number of RNPs packaged within the respective whole virions was counted. All A/WSN virions examined (*n* = 50) were found to have packaged all eight RNPs arranged in the “7+1” pattern (Fig. 4A), which is largely consistent with the results of TEM analysis of 110-nm-thick ultrathin sections (Fig. 1A). Similarly, for the other strains examined, most of the 3-D reconstructed virions packaged all eight RNPs: 91% for A/Yokosuka (*n* = 11), 86% for B/Lee (*n* = 49), and 80% for B/Yokosuka (*n* = 10). These results suggest that virions containing less than eight RNPs are in the minority. Indeed, virions lacking RNPs accounted for no more than 20% of the 3-D reconstructed virions (Fig. 4). Because no virions containing less than five RNPs were observed among the tested strains, the existence of such virions remains unknown.

## DISCUSSION

It is generally believed that influenza A viruses selectively package their eight different RNPs within their virions (21–25). However, some virological studies have reported that most virions are in fact SI virions and that this is likely due to incorrect packaging and a lack of RNP(s) (27, 29–31). To directly demonstrate how many RNPs are packaged into different virions, we used electron microscopy and confirmed that both influenza A and B virions usually package eight RNPs, arranged in the “7+1” pattern. Importantly, we also provide the first direct evidence that some virions are indeed deficient in one or more RNP(s) and that the proportion of virions lacking RNP(s) is dependent on the virus strain. These results are consistent with the current selective genome-packaging model but suggest that this mechanism of influenza A and B virus packaging is more flexible than previously thought.

Using STEM tomography, we directly counted the number of RNPs packaged within 3-D reconstructed virions and revealed that only a small proportion of virions (at most 20%) packaged less than eight RNPs. Overall, these results show that clinically isolated strains of both influenza A and B viruses have a greater tendency to package less than eight RNPs than do their laboratory counterparts (Fig. 1 and 2). Although we did not determine the infectivity of the viruses in this study, a virion with less than eight RNPs would not be able to undergo the replication cycle by itself, suggesting that those virions share a feature similar to SI virions. Previous studies determined the proportion of SI virions by analyzing the viral protein expression in infected cells. Brooke et al. (30) performed flow cytometric analysis and reported that 53% of A/PR8 virus-infected cells failed to simultaneously express hemagglutinin (HA), neuraminidase (NA), nucleoprotein (NP), and NS1 proteins, suggesting that most of these influenza A virions were SI virions (30). An immunofluorescence assay of A/WSN virus-infected cells by Martin and Helenius revealed that approximately 5% of NP-expressing cells failed to express either HA or M1 (29). Failure of viral protein expression could be due to not only the failure of vRNA packaging into virions but could also reflect the uncoating and inefficient nuclear transport of RNPs or lack of vRNA transcription and/or viral protein synthesis, even though eight RNPs were packaged in virions (32). Another possible mechanism for the production of SI virions is that the virions package eight RNPs but incorporate two or more of the same vRNPs, resulting in some vRNPs not being represented. However, a previous study showed that vRNAs that possess the same genome-packaging signal but encode different reporter genes compete with each other, suggesting that multiple copies of the same vRNPs would not be efficiently incorporated into a virion under normal circumstances (33). In fact, we showed that the proportion of virions that package a complete set of RNPs for A/WSN was 100%, suggesting that some virions with eight RNPs could be semiinfectious due to packaging nonfunctional vRNA segment (e.g., those possessing mutations leading to nonfunctional proteins). The presence of SI virions with eight RNPs may be what caused the difference in the proportion of SI virions as previously reported (29, 30) and virions with less than eight RNPs determined in this study (Fig. 4A). It should be noted that the proportion of virions that package eight RNPs and less than eight RNPs may vary by host cell type and also at different time points after infection. Further studies are needed to determine the factors that affect the number of the RNPs that are packaged within progeny virions.

Taken together, our results essentially support the selective packaging model, in which eight different vRNAs are generally packaged into virions. As expected, the arrangement of eight RNPs in the typical “7+1” pattern was observed in all influenza A and B virions examined (Fig. 1 and 2). Although the number of RNPs observed within a single whole virion varied from five to eight, the overall configurations of the RNPs inside the virions were consistent: one RNP at the center surrounded by the remaining RNPs (Fig. 3B, D, F, and H; see Fig. S2B, S2D, and S2F in the supplemental material). Previous reverse genetics studies have demonstrated that mutations or deletions in a packaging signal sequence of a certain vRNA can disrupt the packaging of the other vRNAs into the virions or virus-like particles (VLPs), suggesting functional interactions among vRNAs via a genome-bundling signal—which ensures the incorporation of the complete set of eight vRNAs into the virions (20)—during the genome-packaging process (26). Deletion of the genome-packaging signals in the PB2 vRNA had the strongest impact on the packaging efficiency of other vRNAs into VLPs (14), suggesting the importance of the PB2 vRNA in genome packaging. A detailed ultrastructural analysis of the eight well-organized RNPs within virions revealed physical interactions among the central and peripheral RNPs via as-yet-unidentified string-like structures (22). Considering these specific interactions, our data suggest that the central RNP plays an important role in recruitment and packaging via interactions with the peripheral RNPs. Although the central RNP (vRNA) has not yet been identified, the PB2 vRNA might be a potential candidate (14, 22). Further studies are needed to identify the central RNP (vRNA) among the eight well-organized RNPs as well as hierarchy among vRNPs in packaging other than the critical role played by the PB2 vRNP.

The production of virions that lack RNP(s) may reflect the “flexibility” in the interaction between vRNAs during the genome-packaging process. At first glance, production of virions with less than eight RNPs would seem to be a disadvantage for a virus; however, such flexibility would enable the progeny virions to package vRNAs from different influenza strains, leading to the production of reassortants and thus promoting evolution and diversity. If the selectivity of the eight different vRNAs was rigid and exclusively determined on the basis of conserved nucleotide sequences in genome-packaging signals, the chance of genome reassortment between different strains would be restricted to the certain factors such as high rate of coinfection by different strains. Indeed, a recent study demonstrated that genome reassortment is enhanced by the presence of SI virions that deliver an incomplete genome set (31). Although a single SI virion cannot produce progeny in a cell on its own, it can achieve infectivity via complementation through coinfection with other SI virions. Thus, our results support the idea that flexibility in vRNA interactions may enhance the genome diversity of influenza viruses, even though this property comes at the risk of potential failure of integral vRNA packaging into virions.

Here, we demonstrated that the selective genome-packaging mechanism of eight vRNAs is maintained in influenza A and B viruses, indicating that this mechanism has been conserved as a minimum requirement for continual virus replication and species survival. We also provide the first direct evidence for the presence of virions that package less than eight RNPs and further evidence that most virions package all eight RNPs. Additional studies are required to reveal the genome-packaging mechanism as well as the specific roles of virions with less than eight RNPs. Such studies would contribute to the development of a deeper understanding of how pandemic influenza viruses emerge.

## MATERIALS AND METHODS

### Cells and viruses.

The Madin-Darby canine kidney (MDCK) cells used in this study were obtained from Robert Webster, St. Jude Children’s Research Hospital, Memphis, TN, and were maintained in minimal essential medium (Gibco) with 5% newborn calf serum. The stocks of A/WSN/33 (A/WSN), A/Puerto Rico/8 (A/PR8), B/Lee/40 (B/Lee), and B/Hong Kong/8/73 (B/HK) were prepared by using reverse genetics as previously described (34). A/Yokosuka/UT-Y291/11 (A/Yokosuka), A/Tokyo/UT-IMS6-1/2013 (A/Tokyo), B/Yokosuka/UT-Y231/11 (B/Yokosuka), and B/Yokohama/UT2086/03 (B/Yokohama), which were clinically isolated from humans, were passaged three times in MDCK cells.

### Virus infection.

The cells were infected with each virus strain at a multiplicity of infection (MOI) of 10 and were fixed at 10 to 16 h postinfection (hpi) as previously described (21).

### Quantification of the number of packaged RNPs in virions by use of TEM.

Chemically fixed samples were prepared as previously described (21). Ultrathin (110-nm-thick) sections were stained with 2% uranyl acetate and Reynold’s lead. The areas of interest were chosen randomly based on where budding virions and infected cells were observed. The images were then acquired with a Tecnai F20 TEM (FEI Company, Eindhoven, the Netherlands) at 200 kV. The number of RNPs in each virion was determined by counting the number of RNPs observed only within transversely sectioned virions. This was because the number of RNPs within the longitudinally sectioned virions could not be counted due to overlapping projection images of other RNPs within the virions. Empty virions were not considered in this study because the rate of empty virions is dependent on strain-specific morphology; filamentous virions appear to be mostly empty when they are transversely sectioned (21). Approximately 100 virions were observed for each virus strain.

### Quantification of the number of packaged RNPs in virions by use of STEM.

Semithin sections (250 nm thick) were prepared from the same plastic block as was used for the ultrathin sections. After the semithin sections were stained with 2% uranyl acetate and Reynold’s solution, both sides of the sections were carbon coated with the VE-2030 vacuum (Vacuum Device, Ibaraki, Japan). Plasma cleaning was performed with a model 1020 plasma cleaner (Fischione Instruments, Export, PA). Single- or dual-axis images of the semithin sections were acquired with a Tecnai F20 field-emission STEM (FEI Company, Eindhoven, the Netherlands) at 200 kV using an annular dark-field detector (Fischione, Export, PA). The digital images were collected with a 2cosθ° increment over a 60° range with a pixel size ranging from 0.25 to 1 nm. The stack of images was then reconstructed by using the simultaneous iterative reconstruction technique and Inspect3D software (FEI Company, Eindhoven, the Netherlands). For the dual-axis tilt series, the *x*- and *y*-axis tilt series were reconstructed by using Inspect3D software (FEI Company, Eindhoven, the Netherlands). The models of the RNP complexes within the virions were created with the Avizo 6.2 image-processing package (Visualization Science Group, Burlington, MA) as previously described (22).

## SUPPLEMENTAL MATERIAL

Figure S1  Schematic diagram of virions within an ultrathin section and their correlative images by using TEM. Because whole virions are not always contained within 110-nm-thick ultrathin sections, the number of RNPs observed within sectioned virions may differ depending on where a virion is located within the section. (A) When a virion is located entirely within a section, the number of RNPs observed within the virion on the projected images accurately reflects the number of RNPs packaged. (B) When the top part of the virion is partially contained within a section, the number of RNPs within the virion on the projection images may still represent the actual number of packaged RNPs within the virion, given that RNPs are always found at one end of the virion. (C) If the virion, especially the bottom part of the virion, is partially contained within the section, the projected image may not accurately represent the real number of RNPs packaged within the virion. This is possible because the RNPs are found at one end of the virion and the RNPs differ in length. Download Figure S1, TIF file, 8.5 MB

Figure S2  Influenza A and B viruses package eight RNPs in the “7+1” configuration. For each virus strain, 250-nm-thick semithin sections were prepared from the same samples as those examined by using TEM (Fig. 1 and 2). Then, 3-D structures of the virions were reconstructed by using STEM tomography. Digital slices of reconstructed virions for A/Yokosuka (A and B), B/Lee (C and D), and B/Yokosuka (E and F) are shown from the top (top left panel) to the bottom (bottom left panel). (B, D, F, and H) Model figures of the RNPs packaged within the virions from the top (right) and side (left) view. Scale bar, 75 nm. Download Figure S2, TIF file, 8.2 MB

Movie S1A  Movie of the model RNPs within the reconstructed A/WSN virion shown in Fig. 3. Download Movie S1A, AVI file, 9.3 MB

Movie S1B  Movie of the model RNPs within the reconstructed B/Lee virion shown in Fig. 3. Download Movie S1B, AVI file, 8 MB

Movie S1C  Movie of the model RNPs within the reconstructed B/Lee virion shown in Fig. 3. Download Movie S1C, AVI file, 5.6 MB

Movie S1D  Movie of the model RNPs within the reconstructed A/Yokosuka virion shown in Fig. 3. Download Movie S1D, AVI file, 9.1 MB

## References

[1] Palese P, Shaw ML 2007 Orthomyxoviridae: the viruses and their replication, p 1647–1689. *In* Knipe DM, Howley PM, Griffin DE, Lamb RA, Martin MA, Roizman B, Strause SE (ed), Fields virology, 5th ed. Lippincott Williams & Wilkins, Philadelphia, PA.

[2] Sugita Y, Sagara H, Noda T, Kawaoka Y 2013 Configuration of viral ribonucleoprotein complexes within the influenza A virion. J Virol 87:12879–12884. doi:10.1128/JVI.02096-13. 24067952 PMC3838143

[3] Arranz R, Coloma R, Chichón FJ, Conesa JJ, Carrascosa JL, Valpuesta JM, Ortín J, Martín-Benito J 2012 The structure of native influenza virion ribonucleoproteins. Science 338:1634–1637. doi:10.1126/science.1228172. 23180776

[4] Moeller A, Kirchdoerfer RN, Potter CS, Carragher B, Wilson IA 2012 Organization of the influenza virus replication machinery. Science 338:1631–1634. doi:10.1126/science.1227270. 23180774 PMC3578580

[5] Compans RW, Content J, Duesberg PH 1972 Structure of the ribonucleoprotein of influenza virus. J Virol 10:795–800. 4117350 10.1128/jvi.10.4.795-800.1972 PMC356535

[6] Neumann G, Noda T, Kawaoka Y 2009 Emergence and pandemic potential of swine-origin H1N1 influenza virus. Nature 459:931–939. doi:10.1038/nature08157. 19525932 PMC2873852

[7] Garten RJ, Davis CT, Russell CA, Shu B, Lindstrom S, Balish A, Sessions WM, Xu X, Skepner E, Deyde V, Okomo-Adhiambo M, Gubareva L, Barnes J, Smith CB, Emery SL, Hillman MJ, Rivailler P, Smagala J, de Graaf M, Burke DF, Fouchier RA, Pappas C, Alpuche-Aranda CM, López-Gatell H, Olivera H, López I, Myers CA, Faix D, Blair PJ, Yu C, Keene KM, Dotson PD, Boxrud D, Sambol AR, Abid SH, St George K, Bannerman T, Moore AL, Stringer DJ, Blevins P, Demmler-Harrison GJ, Ginsberg M, Kriner P, Waterman S, Smole S, Guevara HF, Belongia EA, Clark PA, Beatrice ST, Donis R, Katz J, et al. 2009 Antigenic and genetic characteristics of swine-origin 2009 A(H1N1) influenza viruses circulating in humans. Science 325:197–201. doi:10.1126/science.1176225. 19465683 PMC3250984

[8] Smith GJ, Vijaykrishna D, Bahl J, Lycett SJ, Worobey M, Pybus OG, Ma SK, Cheung CL, Raghwani J, Bhatt S, Peiris JS, Guan Y, Rambaut A 2009 Origins and evolutionary genomics of the 2009 swine-origin H1N1 influenza A epidemic. Nature 459:1122–1125. doi:10.1038/nature08182. 19516283

[9] Hutchinson EC, von Kirchbach JC, Gog JR, Digard P 2010 Genome packaging in influenza A virus. J Gen Virol 91:313–328. doi:10.1099/vir.0.017608-0. 19955561

[10] Gerber M, Isel C, Moules V, Marquet R 2014 Selective packaging of the influenza A genome and consequences for genetic reassortment. Trends Microbiol 22:446–455. doi:10.1016/j.tim.2014.04.001. 24798745

[11] Fujii Y, Goto H, Watanabe T, Yoshida T, Kawaoka Y 2003 Selective incorporation of influenza virus RNA segments into virions. Proc Natl Acad Sci U S A 100:2002–2007. doi:10.1073/pnas.0437772100. 12574509 PMC149948

[12] Liang Y, Hong Y, Parslow TG 2005 *cis*-Acting packaging signals in the influenza virus PB1, PB2, and PA genomic RNA segments. J Virol 79:10348–10355. doi:10.1128/JVI.79.16.10348-10355.2005. 16051827 PMC1182667

[13] Liang Y, Huang T, Ly H, Parslow TG, Liang Y 2008 Mutational analyses of packaging signals in influenza virus PA, PB1, and PB2 genomic RNA segments. J Virol 82:229–236. doi:10.1128/JVI.01541-07. 17959657 PMC2224372

[14] Muramoto Y, Takada A, Fujii K, Noda T, Iwatsuki-Horimoto K, Watanabe S, Horimoto T, Kida H, Kawaoka Y 2006 Hierarchy among viral RNA (vRNA) segments in their role in vRNA incorporation into influenza A virions. J Virol 80:2318–2325. doi:10.1128/JVI.80.5.2318-2325.2006. 16474138 PMC1395381

[15] Marsh GA, Hatami R, Palese P 2007 Specific residues of the influenza A virus hemagglutinin viral RNA are important for efficient packaging into budding virions. J Virol 81:9727–9736. doi:10.1128/JVI.01144-07. 17634232 PMC2045411

[16] Watanabe T, Watanabe S, Noda T, Fujii Y, Kawaoka Y 2003 Exploitation of nucleic acid packaging signals to generate a novel influenza virus-based vector stably expressing two foreign genes. J Virol 77:10575–10583. doi:10.1128/JVI.77.19.10575-10583.2003. 12970442 PMC228515

[17] Ozawa M, Fujii K, Muramoto Y, Yamada S, Yamayoshi S, Takada A, Goto H, Horimoto T, Kawaoka Y 2007 Contributions of two nuclear localization signals of influenza A virus nucleoprotein to viral replication. J Virol 81:30–41. doi:10.1128/JVI.01434-06. 17050598 PMC1797272

[18] Fujii K, Ozawa M, Iwatsuki-Horimoto K, Horimoto T, Kawaoka Y 2009 Incorporation of influenza A virus genome segments does not absolutely require wild-type sequences. J Gen Virol 90:1734–1740. doi:10.1099/vir.0.010355-0. 19297607 PMC2731938

[19] Fujii K, Fujii Y, Noda T, Muramoto Y, Watanabe T, Takada A, Goto H, Horimoto T, Kawaoka Y 2005 Importance of both the coding and the segment-specific noncoding regions of the influenza A virus NS segment for its efficient incorporation into virions. J Virol 79:3766–3774. doi:10.1128/JVI.79.6.3766-3774.2005. 15731270 PMC1075679

[20] Goto H, Muramoto Y, Noda T, Kawaoka Y 2013 The genome-packaging signal of the influenza A virus genome comprises a genome incorporation signal and a genome-bundling signal. J Virol 87:11316–11322. doi:10.1128/JVI.01301-13. 23926345 PMC3807325

[21] Noda T, Sagara H, Yen A, Takada A, Kida H, Cheng RH, Kawaoka Y 2006 Architecture of ribonucleoprotein complexes in influenza A virus particles. Nature 439:490–492. doi:10.1038/nature04378. 16437116

[22] Noda T, Sugita Y, Aoyama K, Hirase A, Kawakami E, Miyazawa A, Sagara H, Kawaoka Y 2012 Three-dimensional analysis of ribonucleoprotein complexes in influenza A virus. Nature Commun 3:639. doi:10.1038/ncomms1647. 22273677 PMC3272569

[23] Gavazzi C, Isel C, Fournier E, Moules V, Cavalier A, Thomas D, Lina B, Marquet R 2013 An *in vitro* network of intermolecular interactions between viral RNA segments of an avian H5N2 influenza A virus: comparison with a human H3N2 virus. Nucleic Acids Res 41:1241–1254. doi:10.1093/nar/gks1181. 23221636 PMC3553942

[24] Fournier E, Moules V, Essere B, Paillart J-C, Sirbat J-D, Isel C, Cavalier A, Rolland J-P, Thomas D, Lina B, Marquet R 2012 A supramolecular assembly formed by influenza A virus genomic RNA segments. Nucleic Acids Res 40:2197–2209. doi:10.1093/nar/gkr985. 22075989 PMC3300030

[25] Chou YY, Vafabakhsh R, Doğanay S, Gao Q, Ha T, Palese P 2012 One influenza virus particle packages eight unique viral RNAs as shown by FISH analysis. Proc Natl Acad Sci U S A 109:9101–9106. doi:10.1073/pnas.1206069109. 22547828 PMC3384215

[26] Gavazzi C, Yver M, Isel C, Smyth RP, Rosa-Calatrava M, Lina B, Moulès V, Marquet R 2013 A functional sequence-specific interaction between influenza A virus genomic RNA segments. Proc Natl Acad Sci U S A 110:16604–16609. doi:10.1073/pnas.1314419110. 24067651 PMC3799358

[27] Donald HB, Isaacs A 1954 Counts of influenza virus particles. J Gen Microbiol 10:457–464. doi:10.1099/00221287-10-3-457. 13174769

[28] Wei Z, McEvoy M, Razinkov V, Polozova A, Li E, Casas-Finet J, Tous GI, Balu P, Pan AA, Mehta H, Schenerman MA 2007 Biophysical characterization of influenza virus subpopulations using field flow fractionation and multiangle light scattering: correlation of particle counts, size distribution and infectivity. J Virol Methods 144:122–132. doi:10.1016/j.jviromet.2007.04.008. 17586059

[29] Martin K, Helenius A 1991 Nuclear transport of influenza virus ribonucleoproteins: the viral matrix protein (M1) promotes export and inhibits import. Cell 67:117–130. doi:10.1016/0092-8674(91)90576-K. 1913813

[30] Brooke CB, Ince WL, Wrammert J, Ahmed R, Wilson PC, Bennink JR, Yewdell JW 2013 Most influenza A virions fail to express at least one essential viral protein. J Virol 87:3155–3162. doi:10.1128/JVI.02284-12. 23283949 PMC3592173

[31] Fonville JM, Marshall N, Tao H, Steel J, Lowen AC 2015 Influenza virus reassortment is enhanced by semi-infectious particles but can be suppressed by defective interfering particles. PLoS Pathog 11:e1005204. doi:10.1371/journal.ppat.1005204. 26440404 PMC4595279

[32] Brooke CB, Ince WL, Wei J, Bennink JR, Yewdell JW 2014 Influenza A virus nucleoprotein selectively decreases neuraminidase gene-segment packaging while enhancing viral fitness and transmissibility. Proc Natl Acad Sci U S A 111:16854–16859. doi:10.1073/pnas.1415396111. 25385602 PMC4250133

[33] Inagaki A, Goto H, Kakugawa S, Ozawa M, Kawaoka Y 2012 Competitive incorporation of homologous gene segments of influenza A virus into virions. J Virol 86:10200–10202. doi:10.1128/JVI.01204-12. 22740412 PMC3446617

[34] Neumann G, Watanabe T, Ito H, Watanabe S, Goto H, Gao P, Hughes M, Perez DR, Donis R, Hoffmann E, Hobom G, Kawaoka Y 1999 Generation of influenza A viruses entirely from cloned cDNAs. Proc Natl Acad Sci U S A 96:9345–9350. doi:10.1073/pnas.96.16.9345. 10430945 PMC17785

