# Supplementary figures and images for: Complete and Incomplete Genome Packaging of Influenza A and B Viruses
Source: mBio. 2016 Sep 6;7(5):e01248-16. doi: 10.1128/mBio.01248-16 (PMC5013298; doi:10.1128/mBio.01248-16)

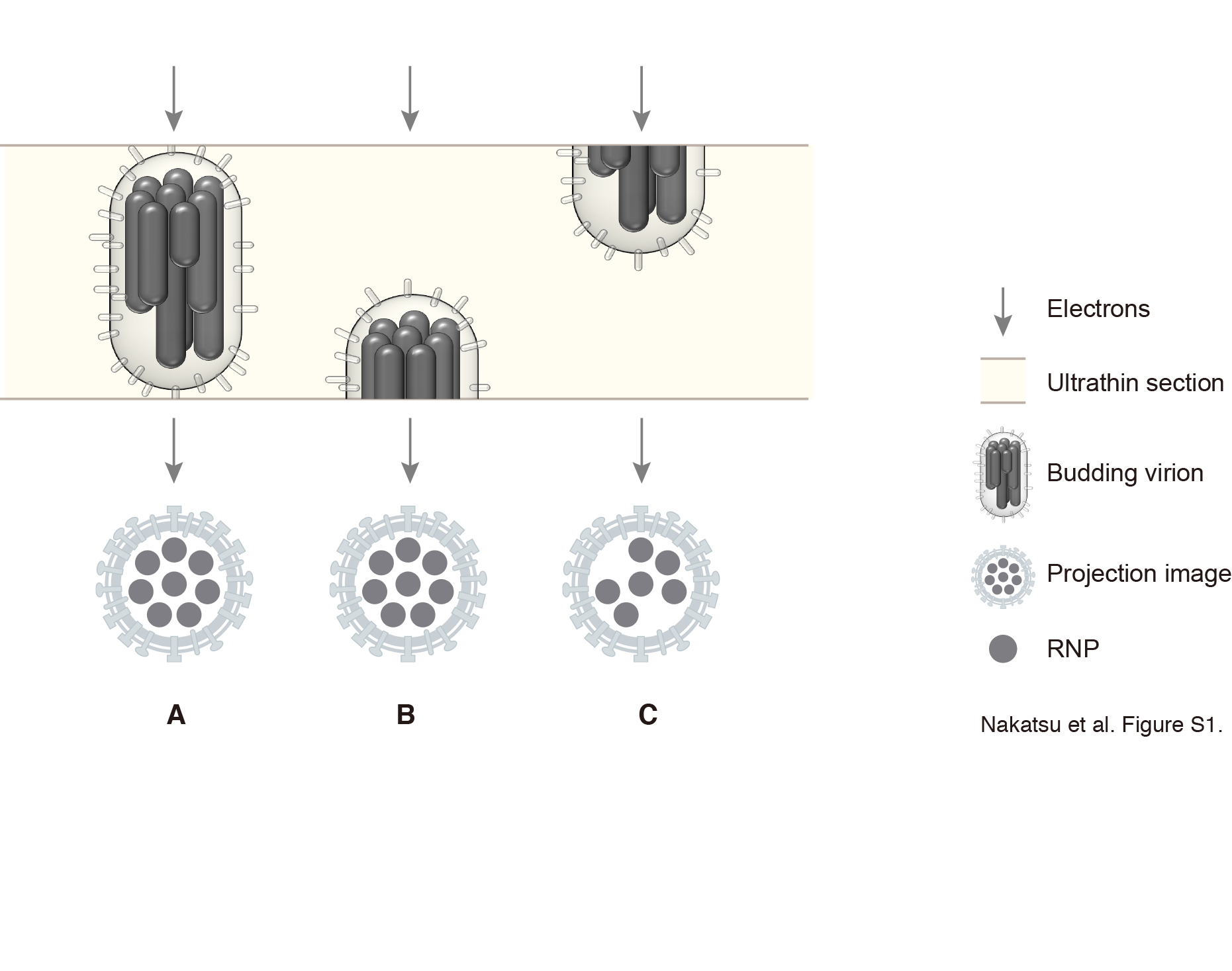

Supplement: Figure S1 — Schematic diagram of virions within an ultrathin section and their correlative images by using TEM. Because whole virions are not always contained within 110-nm-thick ultrathin sections, the number of RNPs observed within sectioned virions may differ depending on where a virion is located within the section. (A) When a virion is located entirely within a section, the number of RNPs observed within the virion on the projected images accurately reflects the number of RNPs packaged. (B) When the top part of the virion is partially contained within a section, the number of RNPs within the virion on the projection images may still represent the actual number of packaged RNPs within the virion, given that RNPs are always found at one end of the virion. (C) If the virion, especially the bottom part of the virion, is partially contained within the section, the projected image may not accurately represent the real number of RNPs packaged within the virion. This is possible because the RNPs are found at one end of the virion and the RNPs differ in length. Download [file mbo004162975sf1.tif]

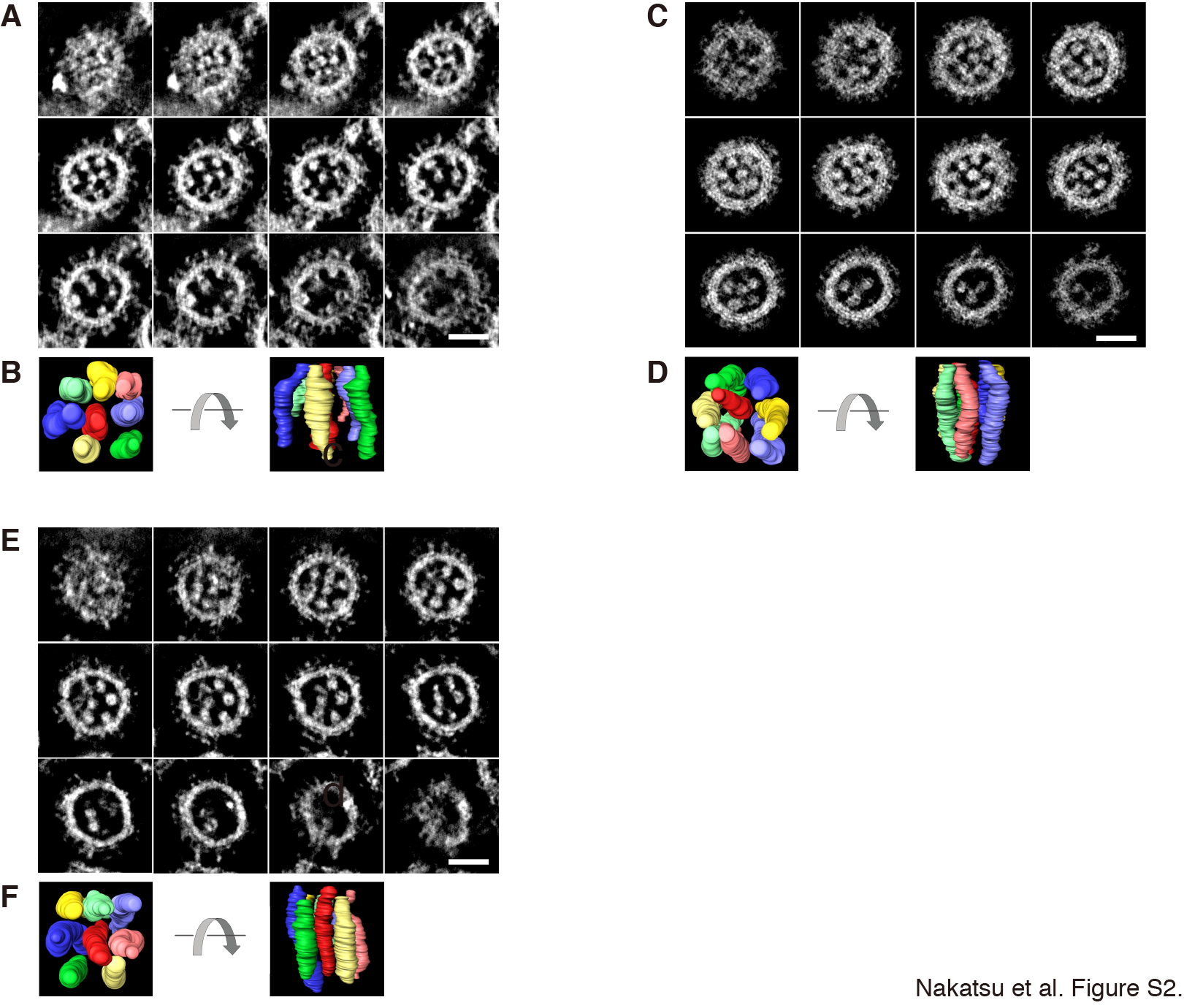

Supplement: Figure S2 — Influenza A and B viruses package eight RNPs in the “7+1” configuration. For each virus strain, 250-nm-thick semithin sections were prepared from the same samples as those examined by using TEM (Fig. 1 and 2). Then, 3-D structures of the virions were reconstructed by using STEM tomography. Digital slices of reconstructed virions for A/Yokosuka (A and B), B/Lee (C and D), and B/Yokosuka (E and F) are shown from the top (top left panel) to the bottom (bottom left panel). (B, D, F, and H) Model figures of the RNPs packaged within the virions from the top (right) and side (left) view. Scale bar, 75 nm. Download [file mbo004162975sf2.tif]
